# Using Pericardium Allograft in Closing Tracheal Fistula after Removing Tracheotomy Tube

**Published:** 2017-05

**Authors:** Shahrokh Attarian, Fereidoon Sirati

**Affiliations:** 1 Department of Plastic Surgery, Zanajan University of Medical Sciences, Zanjan, Iran;; 2 Department of Surgery, Tehran University of Medical Sciences, Tehran, Iran

**Keywords:** Pericardium allograft, Tracheal fistula, Tracheotomy tube

**DEAR EDITOR**

Tracheotomy is used to keep and control airways open and protect them in head and neck operations.^1^^-^^3^ Tracheotomy is opening a direct airway through an incision in the trachea observed as a stoma in the lower throat and it is usually performed in serious head and neck traumas, tumors, bleedings and cases that intubation with endotracheal tube is not possible. This method has several advantages for the patient, including suction of lung secretions, decreasing respiratory dead space and application of positive pressure on the lungs. Also tracheotomy prevents aspiration of mouth and stomach secretions in unconscious or paralyzed patients. The location of tracheotomy incision is trachea fourth cartilage ring under cricoids. Tracheotomy tube is inserted through a small incision on the neck between two cartilage rings into the trachea. The resulting open wound is prone to infections.^4^^,^^5^

To perform tracheotomy first a cuffed tube and after about 1.5 months and non-cuffed tube is used. Cuff is a balloon like addition on the tracheotomy tube that enters the trachea and can be filled or emptied through a valve by syringes outside the trachea. Cuff prevents choking due to being unable to swallow food or secretions in fully or semi unconscious patients. After treatment is complete and ensuring that the upper tracheotomy tube airways are open, the tube is removed slowly and step by step from the patient’s trachea. The wound from tracheotomy closes on its own within 1 to 2 months but may have the following side effects: Skin dimpling, neck deformity, obstruction in respiratory tract, respiratory stridor, vocal complications, lung and airway infections. neck deformity is repaired by a separate operation with platysma flop and zplasty.^4^^,^^5^

Our suggested method is using pericardium allograft or facial allograft in repairing the trachea trauma.^4^^,^^5^ Patients with enough tissue to cover the lesion, their own tissues are used as autograft. But the main problem is for patients without sufficient tissues. One of the best options is using allograft tissues from another person. In most advance medical centers, allograft tissues from brain dead individuals or corpses are used. Bone, cartilage, tendon and skin donors must be free from diseases. Therefore it is necessary to screen donors to prevent future medical complications. Allograft is supplied from tissue banks. Countries such as the Netherlands have a tissue bank that supplies even the whole European Union.^6^

In transplant of organs such as kidney or cornea, the donated organ must be used immediately but allograft transplant can be frozen and preserved. Allograft can be processed and preserved for month’s even years after harvesting. Of course this requires physical space and specialized team to remove tissues from brain dead individuals or corpses. In this method only a thin layer of donor’s tissue is removed and transplanted into patients requiring allograft. The intended graft is removed from corpses of young individuals age 15 to 45 died from accidents whose death occurred not more than 12 hours.^7^

After preparing lower body organs, an incision is made in the groin and 10mm blood sample is taken from the femoral vein and the serum is sent to be screened for diseases such as AIDS and hepatitis by Iran Blood Transfusion Organization. After ensuring the health of the intended tissues and washing them by physiology serums, various cultures are mad and the samples are prepared by lyophilization through gamma radiation, cell washing and also sterilization into dry, cell less, sterile ready to use packs. For transplant purposes the allograft tissues are screened for viral and bacterial infections and only infection free samples are selected.^8^^,^^9^

Also after culturing cells they are evaluated for bacterial and Mycoplasma contamination and Karyotype is performed to assess changes in the chromosomes of the cultured cells in order to have the utmost healthy transplant cells. In this part a successful case of allograft used for restoring trachea trauma is introduced: The patient is male, 25 years, who suffered lefrot III fractures, various lesions and laceration of facial soft tissues due to being in a motor vehicle accident (motor cycle). Because of the extensive fractures and traumas to the face and jaws the patient underwent tracheotomy. After completion of jaw, nose and mouth surgery and soft tissue controlling lower airways and lungs, tracheotomy tube was removed and intubation with spiral tube was conducted. 

Afterwards, our operation that replaced the normal procedure was performed to preserve the normal look of the trachea and trachea anatomy by edge to edge repair with pericardium allograft size of 5 cm×5 cm with Monocryl 0-4 thread and zplasy restoration of skin and muscles covering the area. The patient’s respiration was controlled by an anesthesiologist and the patient was extubated carefully. Also the patient’s vital signs, breathing properties and lung sounds, blood gases and oxygen saturation after the operation was monitored and all were deemed satisfactory. The patient was released after 24 hours. After the operation subcutaneous no emphysema repair, vocal and repair complications was observed in the patient. The area had no dimpling and the scar was removed by fractional CO2 laser. The fractional CO2 laser used had the following specifications: Power=3w, with scanner and right to left; Overlap=1, distance=1.

In order to quicken the treatment defecting cartilage with a facial allograft of high strength such as pericardium allograft in a tight and edge to edge method can be used in order to achieve better results in a shorter amount of time. Considering that the lesion scar, physical shape, trachea and lung function and the patient’s psychological complication is removed more quickly in this method of operation, allografts being numerous and accessible and the operation itself being cheap, simple and without side effects, it is therefore prudent not to leave the lesion from tracheotomy to close on its own and use this surgery to achieve a better treatment result.

## CONFLICT OF INTEREST

The authors declare no conflict of interest.
